# Player-Character Relationship and Game Satisfaction in Narrative Game: Focus on Player Experience of Character Switch in *The Last of Us Part II*

**DOI:** 10.3389/fpsyg.2021.709926

**Published:** 2021-09-27

**Authors:** Valérie Erb, Seyeon Lee, Young Yim Doh

**Affiliations:** Games and Life Lab, Graduate School of Culture Technology, Korea Advanced Institute of Science and Technology (KAIST), Daejeon, South Korea

**Keywords:** player character, player-character relationship, game satisfaction, player experience, *The Last of Us*, video games

## Abstract

While player characters (PCs) are the key element in engaging players in narrative games, the experience and relationship of the player with the PC have received scarce attention from the perspective of the subjective player experience. The diversity of players and the importance of the PC in the game suggests meaningful connections between how players relate to their PC and the resulting satisfaction with the game. We, therefore, investigated in this study how the player-character relationship influences satisfaction of the player with the game. We performed semi-structured in-depth interviews with 12 players of *The Last of Us Part II*, a game that has elicited highly polarized reactions in relation to how players responded to a switch of the PC in the game. Through thematic analysis, three themes were found, illustrating the connection between aspects of the player-character relationship and the overall game satisfaction. The themes are “Tolerance of forced character switch”, “Malleability of character image” and “Flexibility of character attachment”. We discuss how those findings should be taken into consideration when designing diverse and meaningful gaming experiences.

## 1. Introduction

Video games are continuously gaining popularity not only as an entertainment medium, but also in fields such as art (Folkerts, 2010; Devine, 2017; Chew and Mitchell, 2020), education (Klopfer et al., 2009), and health (Baranowski et al., 2008; Colder Carras et al., 2018). As the effects of games on players have been mixed (Ferguson, 2007; Prot et al., 2012), it is necessary to gain a deeper understanding of how players engage with games. One of the most important game elements when engaging the player in the game is the player character or avatar (Mallon, 2008; Tychsen et al., 2008; Lankoski, 2011). The player character (PC) is the in-game character which is controlled by the player and through which the player can act in the game world (Vella, 2016). It is useful to distinguish in this study a PC from an avatar. While those terms have been defined inconsistently, an avatar broadly refers to a digital representation of a user (Nowak and Fox, 2018). We understand the main difference therefore as the avatar is to at least some degree customizable, as seen, for example in Massively Multiplayer Online Role-Playing Game (MMORPG) genres, whereas in the narrative genre, the story and features of the PC are to a high degree predefined by the developers.

The majority of research investigating the player experience concerning the PC has been conducted in the context of the MMORPG genre and avatars (Blinka, 2008; Banks, 2015; Banks et al., 2019). This is possibly due to the more obvious involvement of the player in the creation, customization and development of an avatar compared with the mainly predefined PCs. In particular, Jaime Banks has studied the player-avatar relationship in several works (Banks and Bowman, 2013, 2016; Banks, 2015; Banks et al., 2019). In one study (Banks, 2015), she introduces a spectrum of sociality on which the player-avatar relationship can be placed. This spectrum includes four types of player-avatar relationships, namely, seeing the avatar as an object, me, symbiote or social other. The sociality is measured along the dimensions of self-differentiation, emotional intimacy and perceived agency. This model has also been applied to specific game cases (Loyer, 2015). In another study, Banks and Bowman (2016) introduce a metric including ludic and social measures of the player-avatar relationship, which in its refined version (Banks et al., 2019) exhibits the dimensions of relational closeness, anthropomorphic autonomy, critical concern, and sense of control. Since not only in the context of MMORPGs and avatars but also in narrative games and PCs the involvement of the player is central (Drennan et al., 2004; Lankoski, 2011; Vella, 2016), the perspective of the player on PCs is worth further inquiry.

In the context of narrative or story-driven games, the PC takes on a dual role as the controlled figure of the player in the game and a narrative device for the game developers (Lankoski and Bjork, 2008; Jørgensen, 2010). The latter role has received considerable attention in previous research. For example Jørgensen (2010) shows how the game character can be used in different ways by developers to bring across the narrative of the game. Furthermore, Lankoski and Bjork (2008) provide a game design method in which the game-play reflects traits and personality of the character (Lankoski and Bjork, 2008). Lankoski (2011) also studied PC design extensively in relation to player engagement. He proposes a structure of how PCs engage the player in the game, which includes the concepts of recognition, alignment, and allegiance. Recognition is how a player constructs an image of the character based on cues within the game, alignment is the way the information conveyance is structured, and allegiance is related to the moral evaluation of the PC by the player (Lankoski, 2011). While the previously mentioned studies have a theoretical approach in Drennan et al. (2004)'s work focus group interviews with actual players are performed. Their target was to find themes to consider when designing engaging game characters in general, not PCs in particular. The themes were consistency with context, player expectations, social interactions, and consistency with the environment (Drennan et al., 2004). As illustrated, research taking on a design perspective of PCs is extensive; however studies investigating the perspective and experience of the player with PCs are scarce.

While studies on the player-character relationship itself are lacking, there are several works that investigate particular variables of the player-character relationship, such as character attachment (Lewis et al., 2008; Burgess and Jones, 2017; Bopp et al., 2019) or identification (Shaw, 2011; Boudreau, 2012). In Lewis' work, a measurement of character attachment is introduced, encompassing the dimensions of identification/friendship, suspension of disbelief, control, and responsibility (Lewis et al., 2008). Furthermore, the perception of characters in games has been studied by Calleja (2009) who introduces the notion of an alterbiography. An alterbiography describes how the players create their own narrative about the subject in the game through making sense of narrative clues and elements presented in the game (Calleja, 2009). Finally, Burgess and Jones (2017)'s work is interesting as it investigated character attachment and agency in a specific game case study of a controversy surrounding the ending of *Mass Effect 3*. They performed a thematic analysis of online reviews and the resulting themes point to the importance of the player-character relationship in the overall satisfaction of the game. They additionally propose further work to address the found differences across players in character attachment (Burgess and Jones, 2017). While those approaches are valuable, they are limited to the variable in question and do not take into consideration the broader player experience.

One attempt to more comprehensively capture the subjective experience of the player in the creation of a player-character relationship is Ong (2018)'s work where it is attempted to understand the processes through which the relationship with a PC is created (referred to as avatar in the work, but due to no customization or choice option can be understood as PC). However, a more thorough understanding of how the players relate to their PCs is needed, particularly how this relationship influences game satisfaction. One theory that attempts to explain the satisfaction of a media experience in relation to characters in a narrative is disposition theory from drama studies. It states that the affective disposition of a spectator to a character and outcome of that character in the narrative predicts enjoyment of the media experience (Raney, 2004). Such research however does not consider the game context and more complex player-character relationships. Our research thus aims to provide more valuable insights into the nature of the player-character relationship itself and the resulting satisfaction of the player with the game.

We chose to perform a study that aims to explore how the player-character relationship connects to the overall game satisfaction in a narrative game. Particularly, we looked into the context of a game titled *The Last of Us Part II*. In this game, the player is forced to switch PCs midway during the game, to a character who has previously been introduced as an antagonist character and who killed the previous protagonist PC. This game mechanic led to greatly polarized reactions and game satisfaction. It, therefore, seemed an excellent opportunity to explore the link between the player-character relationship and game satisfaction. To gain a deep understanding of the diverse and rich player experience, we chose a qualitative approach, inquiring a sample of a diverse player-base. We performed in-depth semistructured interviews with 12 players of *The Last of Us Part II*. We asked them about their overall game satisfaction, playing experience, and how they engaged with the PCs of the game. Through thematic analysis, we formed themes about the relevant aspects of the player-character relationship in connection to the resulting differences in game satisfaction. The study is structured as follows. In the first section, we introduce methods of the study, such as participants, data analysis, and the game choice. In the next section, we present our results in the form of the themes concerning the player-character relationship and its connection to overall game satisfaction. In the last section, we discuss and conclude our findings including limitations of the study and ideas for future work.

## 2. Methods

### 2.1. Participants and Recruitment

We recruited 12 participants, 7 male and 5 female, with ages ranging from 20 to 40 years, as illustrated in Table 1. Our participants had various nationalities with the majority being South Korean. We purposely recruited participants with diverse nationalities and differing game satisfaction, to capture the variety of possible ways players engaged with the game and PCs. The recruitment took place over posts on university forums, as well as through personal contacts of the researchers, using the snowball principle. The conditions to take part in the study were having played both games *The Last of Us* and *The Last of Us Part II*, or, in case of having quit playing the second game, having watched play-videos of the story until the end. The interviews were compensated with 30 USD or an equivalent in the local currency. The recruitment process and ethical considerations have been approved by the institutional review board of the institute of the authors.

**Table 1:** Study participants.

**Participant**	**Age range**	**Nationality**	**Occupation**	**Game satisfaction**
P1	20–25	Switzerland	Student	Mixed
P2	20–25	Philippines	Student	Positive
P3	20–25	England	Student	Positive
P4	30–35	Ecuador	Student	Mixed
P5	30–35	USA	Accountant	Positive
P6	40–45	South Korea	Professor	Positive
P7	30–35	South Korea	CEO	Positive
P8	30–35	South Korea	Professor	Negative
P9	25–30	South Korea	Office worker	Positive
P10	20–25	South Korea	Student	Negative
P11	20–25	South Korea	Student	Positive
P12	20–25	South Korea	Marketing worker	Negative

### 2.2. Interviews and Thematic Analysis

To explore the richness and diversity of ways to relate to the PCs in the game and to capture all relevant variables related to the game satisfaction, we chose to perform semi-structured in-depth interviews. This approach allowed us to explore different variables of the PC relationship which might be relevant in connection to the game satisfaction and capture the complex context of the playing experiences. The interviews took approximately 1 h and were performed over the online conference system Zoom^1^, due to the Covid-19 pandemic. The interviews were conducted in either English or Korean, with two researchers present, except in one case of P5, where only one researcher was present. After explaining the consent form, the interview was started. First, general questions concerning the background of the participant in playing video games were asked. Next, we inquired about the context and expectations of the players before starting *The Last of Us Part II*. The main part was comprised of questions concerning the relationship and perception of the three main player-characters Joel, Ellie, and Abby such as “How did you like this character, how did you like playing as him/her?” “How would you describe your relationship to that character?”, “Did something change in the way you think about the characters?”. The full question list is provided in the Supplementary Material. The questions sought to elicit responses concerning the impression of the character on the player, their reaction to specific game events (Joel's death, PC switch, finale) and their perceived relationship with the PCs. While we inquired about the experiences of the players in both game parts 1 and 2, we focused more on part 2, since the game satisfaction of this game was more polarized.

The interviews were transcribed and a thematic analysis was performed, using inductive coding in line with Terry et al. (2017). The first author, who can speak in a proficient level of English and advanced level of Korean, coded five interviews in English and one interview in Korean and created an initial coding library. After debriefing and discussion on the coding library, four native Korean coders independently coded the remaining interviews in Korean using the software Nvivo. All coders wrote, shared, and discussed notes. Then, the themes were iteratively revised through discussions between the three authors to triangulate findings and eventually finalized into three themes concerning the player-character relationship and game satisfaction. The quotes from the Korean interviews which are introduced in this study were translated into English.

### 2.3. Game Choice

*The Last of Us Part II* (Naughty Dog, 2020) is a PS4 console game and the continuation of *The Last of Us* (Naughty Dog, 2013). The game can be described as a story-driven action-adventure shooting game, where the player controls mainly two player-characters Ellie and Abby, in the third perspective. The player has no control over the story, which is mainly depicted in cut-scenes and using story-cues in the environment. The player takes on the fighting and moving part of the PC.

The game had very polarized reactions and game satisfaction. On one hand, it received the most ‘Game of the Year’ awards in history (Stedman, 2020; Calvin, 2021) and was praised by critics and many players. On the other hand, players “review bombed” the game on the game-rating platform Metacritic^2^ (Nunneley, 2020), leaving furious reviews. The distribution of user scores on Metacritic shows the polarization concerning this game well, since out of all user reviews on Metacritic only around 5% were mixed, the rest was divided into positive and negative, thus showing slightly more positive reviews^3^. This variety in reactions promised to be illustrative of how players can vary in their engagement with the PC. The focus on the story and complex characters of the game as well as the players' main complaints related to the PCs were indicative that the experiences of the players of this game can provide valuable insights into the different ways players connect to the PCs. For those reasons, we chose this game to perform a case study on how players engage differently with PCs and how this relates to the overall game satisfaction.

The story of the games takes place in a post-apocalyptic America, where half of the population is infected by a disease turning them into zombie-like cannibals. In the first part, the main PC Joel, a man in his forties, has the mission to take the 12 years old Ellie, who happens to be immune to the disease, to a hospital across the country, in an attempt to find a vaccine for the infection. They form a close bond and after he finds out that the operation to develop a vaccine would kill Ellie, he saves her from the operation table, killing several medical staff and allies. He then lies about what happened to Ellie.

The second part takes place 5 years later, where the player mainly controls Ellie who lives peacefully in the safe town of Jackson. Then, the character Abby appears and brutally kills Joel. Ellie goes after Abby with her girlfriend Dina. After killing several of Abby's friends, Ellie faces Abby, who kills and injures some of Ellie's friends. After a cut-scene, the player now controls Abby and learns that Abby's father was the surgeon who Joel (the player) killed in the previous game. Then, the player plays as Abby for the same 3 days until the face-off with Ellie, and continues controlling Abby in fighting Ellie, but after gaining the upper hand in the fight Abby spares Ellie and Dina. Back in Jackson, the player controls Ellie again, who hears about Abby's location and decides to go after her once more, against Dina's will. She finds Abby worn out and captured by another group, and after the last fight, lets her go and returns to an empty home.

## 3. Results

**Figure 1:**
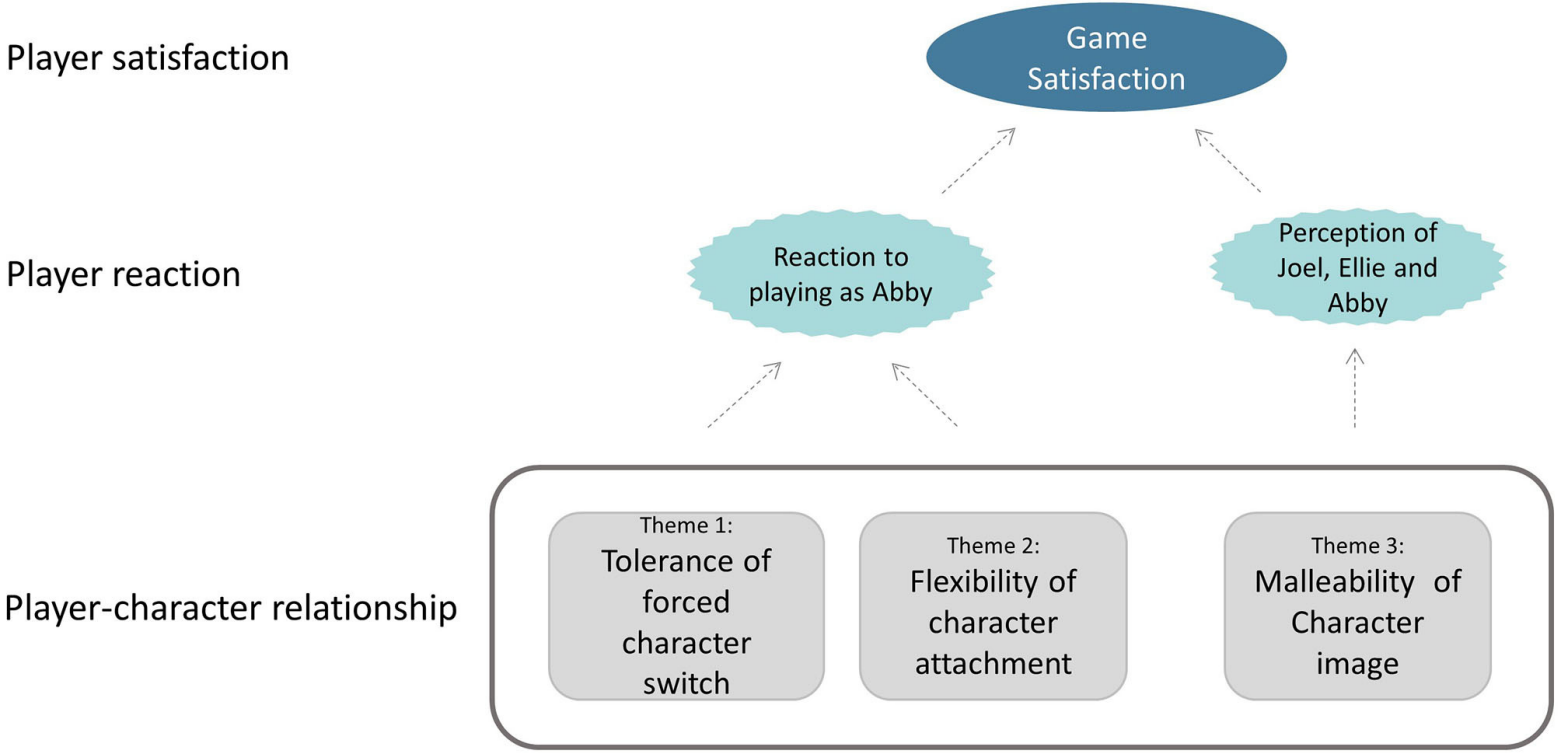
Overview of relationship between game satisfaction, player reactions, and themes concerning player-character relationship.

We illustrate our findings in the following sections. We first illustrate the found differences in overall game satisfaction as well as the more specific player reactions to the game. We then present our themes concerning three aspects of the player-character relationship which were found to be related to the differences in game satisfaction and reactions. Figure 1 illustrates the relationships among the game satisfaction, player reaction, and the themes concerning the player-character relationship.

### 3.1. Differences in Game Satisfaction and Player Reactions

#### 3.1.1. Game Satisfaction

Players varied greatly in whether or how much they were satisfied with *The Last Of Us Part II*. The game satisfaction of the player was often either very high or very low with only a few mixed cases, as illustrated in Table 1. Players who were highly satisfied with the game would describe their experience similar to P5: “*My overall feeling is this is one of the greatest games I've ever played. It's one of the bravest narratives I think I've ever interacted with.”* On the contrary, other players outed their frustration similar to P12: “*I played this emotionally abusive trash game for nothing.”* Two players had mixed feelings about the game and would acknowledge positive and negative aspects like P4 did: “*It was really well done but, it already came with a baggage right, expectations from players (…) it was, narratively speaking, (…) badly structured.”* To understand the differences in the overall game satisfaction better, we looked at more specific player reactions toward the game, as described in the next section.

#### 3.1.2. Player Reactions

The differences in game satisfaction were visible in how the players reacted to the game. There were some clear commonalities in how players who did or did not like the game reacted to certain game events and elements. Those events and elements are (1) the reactions of the players to being forced to play as the Abby character and (2) the perceptions of the players of the different PCs. First, almost all players initially showed great reluctance, confusion, and anger when having to control the Abby character, as she was the character who brutally killed the previous PC Joel. However, the way in which players coped with the continuous obligation to play Abby differed across players. Players with low game satisfaction continuously showed great resistance and either finished the game very reluctantly or all over quit the game. The players who later voiced great appreciation for the game mentioned that they slowly accepted this game-mechanic and started to engage with the new PC. Second, the way players would perceive and describe the three main PCs in the game varied greatly. Players who did not like the game would critique how the actions of the character are illogical, not human-like, and how Abby appears as a tool of the game developer to convey a message. On the other hand, players who greatly valued the game mentioned how the PCs were interesting, complex, and human. This contrast in player's reactions points to underlying factors in how players interacted with the game. In the following, the three themes related to the differences in game satisfaction and player reactions are presented.

### 3.2. Player-Character Relationship

**Figure 2:**
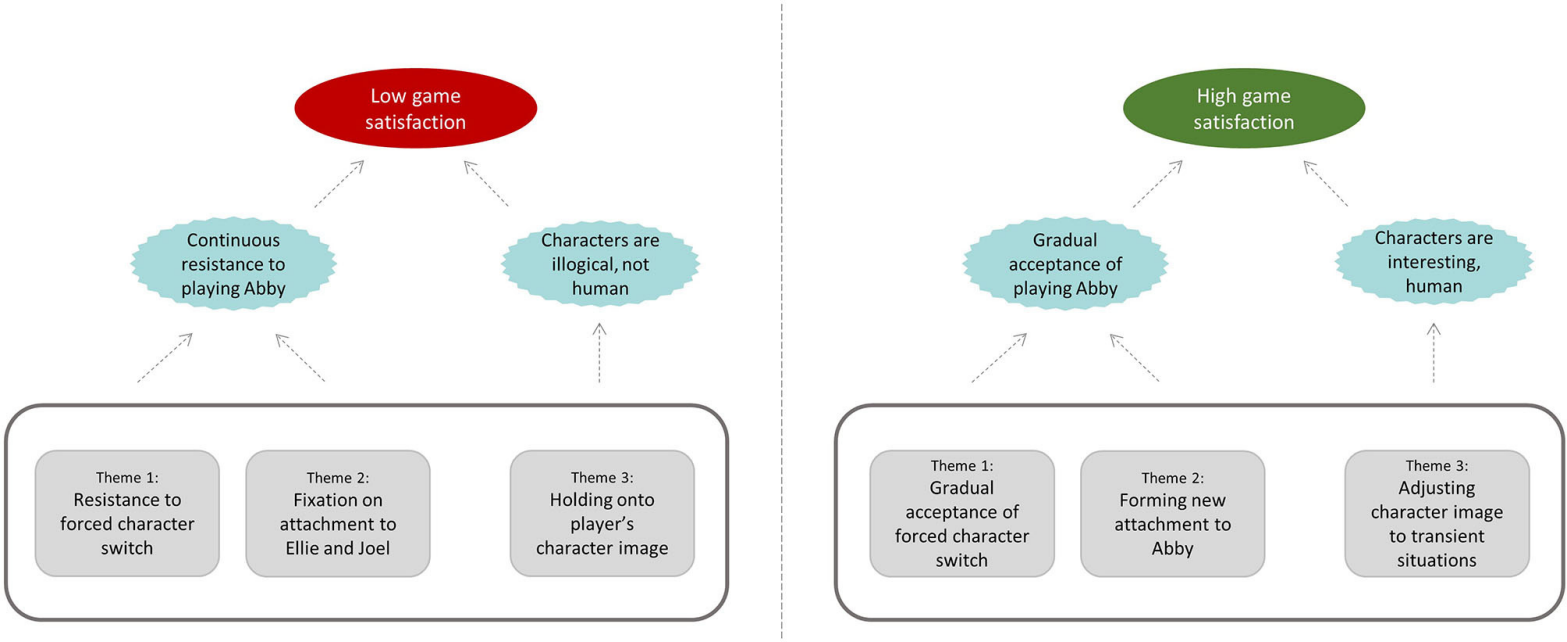
Relationship of themes to high and low game satisfaction.

We now present the three themes which were found in relation to how the participants related to their PCs. We present each theme in the following section illustrating how each aspect of the player-character relationship was related to the players' reactions and overall game satisfaction. Figure 2 illustrates the relationship of the themes in each of low satisfaction and high-satisfaction scenario.

#### 3.2.1. Theme1: Tolerance of Forced Character Switch

The probably most controversial part about *The Last Of Us Part II* is that the player is forced to play as the Abby character midway during the game. The player has no choice but to play as her, to keep progressing in the game. This led to furious reactions in our participants, but not everyone was as resistant to this game mechanic. Some players who had a high game satisfaction valued this forced change of perspective in hindsight, as it enabled them to change their view on the Abby character.

##### 3.2.1.1. Resistance to Forced Character Switch

All but two participants were initially very reluctant to being forced to play as Abby. Of the reluctant participants, five did not appreciate the part as Abby until the end of gameplay. Those players showed a strong reluctance to being forced by the game to play in a way they did not want and having no choice. Particularly, the role of the developer who made them do this was strongly emphasized and the anger was also directly aimed at the development. P8 explains how the Abby character felt like the tool of the developer with which he wants to teach the player something. This created great reluctance in the player so that any engagement with the game was made impossible as P8 stated.

P8: “Every time I played as Abby, it was really tormenting. (…) She was Neil Druckmann's avatar. The character, Abby. So, I keep feeling that Neil Druckmann is trying to convey the game's message through Abby, but it doesn't feel somehow natural like in [part]1, I always felt like he was trying to artificially force it onto me. So I couldn't get any positive feelings for the Abby character even if I wanted to. That's why it was tormenting and exhausting to play her. No sense of immersion at all.”

P8 further mentioned that particularly being forced to directly play Abby made it impossible for him to relate to the character, whereas through other means of story-telling an engagement would, theoretically, have been possible:

P8: “If Abby came up not as a playable character, but always on the other side and the situation would have played out in a cinematic way or so I think I would have been able to relate at least a bit (…) but because the user was forced directly I felt repulsive.”

It can therefore be understood that the forceful play itself, the removal of the agency of the player as a separate element was rejected strongly by the dissatisfied players. The theme is therefore closely related to the continued resistance to playing Abby. This rejection also correlated strongly with the evaluation of the player and eventual satisfaction of the game, as P1 put it: “*When you don't want to do what the game wants you to do it's just not a good game.”* In broader terms, the players did not accept forced gameplay as a narrative tool, as it seemed to go against their understanding of what a game is or should be. This is in contrast to the experiences of the remaining participants of our study, as explained in the following section.

##### 3.2.1.2. Gradual Acceptance of Forced Character Switch

Most of the participants who had high satisfaction with the game were first also very reluctant to being forced to control the Abby character. However, other than the dissatisfied players, the participants started to change their view on the Abby character and the decision to make the player play Abby against their will was appreciated as a bold and effective narrative device, as explained by P5.

P5: “I was furious with Abby up until I had to play as Abby, I mean even through that, the first few scenes of playing as Abby I felt I don't really want to play as her, I hate her, she's awful, she's ruining the life of the person I've really come to enjoy and now I have to play as her. But I think that's genius storytelling. I think it is a risk to tell a story like that to kind of force the player into a perspective that they don't really want to be in. That's gutsy and I appreciate that, not everyone likes that but I really like that. But that way of telling it, by kind of rewinding the clock, loved it I think that's a really smart way of doing it.”

Another participant expressed fascination with how the game medium can be used to make players understand and experience characters differently than in other media, such as literature.

P6: “I was really surprised. Of course in literature there is also attempts like that, but it's really hard to create stories where A kills B and then make them understand that from A's perspective. That's really hard but I thought oh so but through a game that's possible, I often thought that's somehow the game's unique ability. (…) The story isn't like good or bad but it's just those two people's unavoidable encounter and mismatch. How the story was resolved like that was really surprising and great.”

In summary, the forceful deprivation of the agency of the player was not appreciated immediately, by almost any of the participants. However, the satisfied players started to value this forced character play as an effective narrative tool to make them change their minds about the PC, which they considered a meaningful playing experience. For the dissatisfied players, however, their agency seemed a more central part of their gaming experience, which is in direct conflict with the freedom of the developer to use the PC as a story-telling device. Therefore, in their eyes, the developers took not only the agency of the player, but also their game enjoyment to convey their own message. This resulted in strong anger against the developers. An additional factor adding to the players' frustration was that since the game was a sequel to a game without such harsh deprivation of agency, many players did not expect such an experience, and by the time they realized what kind of game *The Last Of Us Part II* is, they had already bought it. The reaction to the forced game play also likely had an influence on the subsequent perception of the PCs by the player, as will be described in Theme 3 and is illustrated in Figure 1.

#### 3.2.2. Theme 2: Flexibility of Character Attachment

This theme concerns how participants formed attachments to the PCs. We show how dissatisfied players fixated on their strong attachment to the PCs, Joel and Ellie, from part one and how other players created an additional attachment to the new PC, Abby. This provided the latter players with a meaningful emotional playing experience which led to high game satisfaction.

##### 3.2.2.1. Fixation on Attachment to Ellie and Joel

While all participants reported having a close attachment to the PCs of the first game, for some of the players this attachment directly interfered with their willingness to form a new attachment to the Abby PC. First, participants reported that they connected to the PCs of the first game through a gradual built up of an emotional bond that was not felt for the Abby character.

P7: “I think it's because I can't ignore the attachment I got for the first game. And as I said before, you can really emphasize with the small emotional changes of Joel who is first just annoyed at Ellie and then gradually starts opening up and getting warmer. But I didn't get that feeling of delicate emotional development from Abby.”

Furthermore, in contrast to satisfied players who would describe their relationship to the character as one of a close friend or movie character, most players who were very dissatisfied with the game mentioned that they immersed or projected themselves into the PC or saw the PC as their alter-ego as described by P12.

P12: “Joel is my alter ego while playing this game. Since I need to occupy that character while playing and since it moves as I do, it's my alter ego which I feel like I can control directly and it gives me the ability to act within the game even though I can't make choices about the story in the game.”

This close connection to the PC could subsequently not be transferred to another PC from another side, which P8 elaborates.

P8: “Anyways since I always have an emotional bond while playing from the perspective of Joel, even if I play Ellie, since Ellie is on Joel's side you can play as her and share that emotional bond. But if you go to the complete opposite side you can't share that emotional bond anymore. Why should I play as her? I don't see any justification for playing as her.”

##### 3.2.2.2. Forming New Attachment to Abby

For the remaining participants, it seemed possible to keep the attachment to Ellie and Joel and still form a new attachment to the Abby character. It was clear that after playing the game the players had an attachment to both main PCs Ellie and Abby at the same time, and this conflicting attachment was one of the core meaningful experiences for which the players reported to appreciate the game for. The degree to which the attachment to Abby was achieved varied; however, a commonality between satisfied players was that they all had some degree of attachment to Abby. P9 explains how this inner conflict of attachment solidifies in the final fight of the game between Ellie and Abby.

P9: “I think this fight might be the real climax of the game. At that time I can really emphasize with Ellie's sadness but I also can roughly understand Abby's life and her changed ideology, so it would be so hard to watch any of the two die”

The ways through which the participants related to the character varied, but frequently mentioned topics were the relationships of Abby with the Yara and Lev character as well as moments where Abby appeared flawed, or vulnerable as illustrated in the following two quotes.

P3: “But then in the second game, once I think the turning point really was once you meet Yara and Lev because that just, brings out Abby's humanity (…) So when then these extra people come and save her and then in turn she goes like she owes them.

P5: “One thing that really struck me as Abby and I love this about the game, is that Abby is terrified of heights. I am also terrified of heights and the game did this so well where they would illustrate her fear when you know she looked down (…) As someone who's afraid of heights I have a similar reaction, so to hear that and to see it depicted so realistically, was another moment of empathy for me and her.”

To summarize, dissatisfied players only had one strong attachment to Joel and Ellie, while the satisfied players had all formed an attachment to the Abby PC to some degree. The former participants immersed or projected themselves strongly into PCs of the first part and were therefore unable to form new attachments while the latter players connected to Abby through her new relationships or hardships.

#### 3.2.3. Theme 3: Malleability of Character Image

The last theme concerns how the players construct and adjust their own mental image of the PC. We found interesting differences in how some participants would see certain actions of the character as “not making sense” and based on that, they criticized the realism or logic of the game, while other players would actively create their own interpretations of why characters acted in the ways they did.

##### 3.2.3.1. Holding Onto Character Image of the Player

A frequent criticism of participants who were disappointed in the game was how illogical the story was, particularly, how the actions of the characters do not make sense. Especially when that character in question was the player's favorite PC, and the actions related to a less positive character representation, this led to strong negative reactions and a negative evaluation of the game as a whole. This is illustrated by P12 who was strongly criticizing the logic of how Joel changed in *The Last Of Us Part II* compared to the first part.

P12: “What I couldn't understand the most was that originally in part 1 Joel was more wary of people than zombies and if he was so strong and able to survive under any circumstances, then the fact that he was too weak to do anything in 2 and gets killed by Abby's group and he was beaten so helplessly to the degree that it made me think it might have been a setting error, made me so angry. (…) It wasn't the Joel I know.”

Note how the last sentence “It wasn't the Joel I know” implies that the representation of the character was deviating from a mental image she had constructed about the PC. Also, inconsistencies in actions of negatively evaluated PCs were criticized, as seen when P8 explains how he got upset by the inconsistent actions of Abby when she spares the NPC Dina.

P8: “Why is she [Abby] acting like this here? If she would act like what is common sense and what she showed us last time, it would have been normal for her to make a massacre ending, but now you don't kill that thing? This feeling of estrangement, based on the first impression she was a terminator who would kill everything, but actually she's a nice person.”

The participant goes on to explain that he felt this inconsistency was due to the developers wanting to bring across their point of Abby actually being a nice character, but this being represented unnaturally in the game.

##### 3.2.3.2. Adjusting Character Image According to Transient Situations

On the other hand we had participants who would flexibly adjust their character image based on new information provided in the game, and create their own interpretation for the underlying reason for the actions of the PC. A great example is P6, who contrary to P12 as discussed above, interpreted Joel's change in behavior as him starting to gain the ability to trust people thanks to Ellie and thereby saw it as character progression.

P6: “In Joel's case, it's just my interpretation, but in Joel's case, it seems that he eventually started to believe in humanity. He believed in humans and built a community. Originally, starting as a very distrustful person, he met Ellie, started to trust Ellie, and while starting to depend on Ellie got to join a community. I understood that this is some kind of growth from his point of view, and that's why I interpret it as him starting to trust people. Of course, looking at the game's realism you can interpret it as Joel just becoming so weird. But Joel now got a deep trust, got to be a person who deeply trusts people.”

P6 continues to explain how he never questions the “logic” or “realism” of a character and his way of approaching game or literary character is that he likes to reason about the underlying motives which move a character.

P6: “Rather than thinking from the outside, in this piece of work this person doesn't make sense, it's not realistic, I'm the type who thinks more about the inside of the person, why did this person have to act in that way? That's why I never really thought things like what's wrong with this person. I don't think those things and there is also a lot of hidden meaning, it's the same with real humans. Why is person A acting like that, that person will have a reason, you don't say that person's action is unrealistic. That's kind of how I see it. (…) I think I treat it like they are real human beings.”

Overall, this can be seen as a stark contrast in how the character image is constructed in players. It is mainly a difference in how players create and adjust their own mental image of the character in contrast to what is represented in the game. Participants who were part of the former type of players were usually the ones with a negative evaluation of the game representation of the game characters, mentioning how the characters do not make sense and exhibited an overall dissatisfaction with the game, while the latter type of players praised the game for it is interesting and complex characters and had high overall game satisfaction. This tendency is likely dependent on individual differences in the player. However, we can also assume that the willingness of the player to create narratives in their mind justifying the characters actions, is strongly influenced by the players' reaction to the forced gameplay and resulting overall attitude toward the game.

## 4. Discussion

### 4.1. Connection to Related Theories

We discuss how our findings relate to the existing literature of player-character relationship and game satisfaction. A simple way to predict the game satisfaction in relation to connections with a media character is disposition theory (Raney, 2004), a theory originally from drama studies which states that the enjoyment of a media experience is based on an individual's affective disposition toward a certain media character and that character's fate within the narrative (Raney, 2004). In the case of *The Last Of Us Part II*, this can explain why the players who only had affection for Ellie and Joel until the end did not have a high enjoyment of the game, since those characters did not have good outcomes in the narrative. However, this theory does not explain why some participants formed an affective disposition to Abby and some did not. Similarly, the concept of allegiance from Lankoski (2011)'s work and its relation to accepting a PC's goal in the narrative can explain the players' reluctance to play as Abby, but not why some players could overcome this reluctance. To better understand those polarities, we must look more closely at the nature of and individual differences in the player-character relationship itself.

Concerning the differences in relating to the PC, we found interesting connections of our findings with the work of Banks (2015). The social spectrum of the player-avatar relationship (PAR) introduced for the context of Avatars in MMORPGs shows some parallels to the context of PCs in *The Last Of Us Part II*, a game with completely predefined characters and story. We found that the participants with a low game satisfaction possibly saw the PC of Joel more as “Me,” while the players with a high satisfaction would see the PC more as a “Social other,” for the following reasons. As illustrated in Theme 1, the disappointed players were very resistant to the forced character switch, since they valued their agency in the game. Even though all players had the same amount of agency in the game, some players likely had a higher perceived agency or valued their agency more. The higher player agency would place the player more toward the “unsocial PAR” side of the spectrum toward “Me” and “Object” (Banks, 2015). In addition, if the player sees the PC as herself, the loss of the agency over “oneself” should be much more shocking and unacceptable than losing agency over a social other.

Furthermore, in Banks and Bowman (2013), the PAR spectrum has been put in relation with the dimensions of character attachment of Lewis et al. (2008). This provides interesting connections to our second theme about the flexibility of character attachment. They show how in “Avatar as Me” identification is high and in “Avatar as Other” sense of care and responsibility is high (Banks and Bowman, 2013). This can explain how both dissatisfied, as well as satisfied players could have a high attachment to the Joel and Ellie character, but this attachment was likely different in nature. Therefore, only the satisfied players who saw the PC as a “Social other” could build an attachment to Abby as an additional “Social other,” while the dissatisfied players could not create a relationship to an additional “Me.” Even though an attachment to an additional “Social other” was possible for several players, the high value in sense of care and responsibility toward Joel and Ellie would suggest helping the PC “get the things it needs in his/her world,” (Banks and Bowman, 2013, p. 2), but those goals were in direct conflict with the goals of the Abby character. This hindered the creation of an attachment but overcoming this inner conflict eventually led to higher game satisfaction.

To understand the differences found in the malleability of the character image, it will be fruitful to consult Calleja (2009)'s work. He explains that players construct a so-called alterbiography, a narrative created during game-play in response to the written narrative and the different game elements. It is the interplay of presented narrative and game mechanics of the designers and the players' subjective sense-making process in the game. The alterbiography is therefore inevitably different from the sole narrative represented in the game and is also different across players. The subject or focalization of the alterbiography can be either a miniature, entity, or the self. Miniature refers to multiple or non-specific entities, and entity and self both refer to single entities in the game, where the differentiation between entity and self is solely dependent on the disposition of the player. The alterbiography is a mental construct that is built up by segments of syntheses, which are the conscious mental efforts of the players to make sense of the game and narrative elements (Calleja, 2009).

The differences we found in our study concerning the creation of an character image might be explained by differences in focalization and the approach of the player to synthesis. For example, as described in section 3.2.3.1, P12 describes her reluctance to accept the changed image of Joel, and she also mentioned earlier in the interview that she saw Joel as her alter-ego, which suggests a focalization on the self and might thereby contribute to a higher reluctance to adjust an alterbiography as it somehow refers to a version of oneself. In addition, P8 outed his frustration about the inconsistent actions of Abby. Other players would bridge this ambiguity through their own interpretations, but the degree to which the player is willing to put effort into forming a synthesis varies and this might also be related to the expectation of the player of how much ambiguity developers should leave in the game. The game satisfaction was, therefore, lower for players who had pertaining conflicts of their existing alterbiography and the presented information in the game.

### 4.2. Implications, Contributions, and Future Work

In our study, we could see that controlling a PC in a narrative game includes more than just the mere mechanical steering of a virtual figure. It can mean to take on and project oneself into a given role in a fixed narrative or closely following a character through their story. The mechanisms involved in the player-character relationships in narrative games are complex and distinct from other genres such as MMORPGs or other media such as film. We could observe in this work how switching the PC can lead to strong reactions, whose causes are likely twofold. First, as our theme 1 illustrates, switching out the PC intrudes the only agency the player has in the game, namely, the control of the PC. Second, the player has to accept to take on the perspective of a new character whose personality, characteristics, and goals might not align with their own preferred outcome of the narrative. Therefore, the reaction to a character switch is highly dependent on how the PC is presented and written. For example, character switches from Joel to Ellie in parts 1 and 2 were quite readily accepted while the new antagonist character Abby initially received strong negative reactions. This illustrates the power of a character switch as a narrative tool, which can bring about highly different outcomes in terms of game satisfaction based on individual differences in the underlying player-character relationship.

Our findings carry several implications about how player-character relationships can be used to convey certain experiences. We found that using a character switch can lead to meaningful and highly valued playing experiences by making the player change their perception about the PC. However, when using the game character as a narrative tool, the following factors should be taken into consideration. First, even though the game does not provide the player with a high degree of direct agency on the story or character creation, players might still highly value their agency of player control. Second, since some players may refer to the PC as a version of themselves and others more as a social other, manipulations of the PC relationship will result in very different player experiences and eventual game satisfaction. Last, the degree to which the game designer's intended picture of a character and the player's alterbiography of that character correspond should never be overestimated, and the willingness of the player to adjust those felt differences is also highly variant.

In this paper, we studied the player-character relationship in a narrative game which includes the switch of a PC. We showed how the experience of the player can differ even in games with a highly predefined narrative and how those differences relate to the game satisfaction. Specifically, we found the themes of “Tolerance of forced character switch,” “Malleability of character image,” and “Flexibility of character attachment.” We illustrated that even though there is a very limited agency in relation to the PC, the players can have perceived agency which they value and are not willing to give up, and there are players who project themselves into the PC, even though the personality of the PC and story are predefined. We also showed how the willingness of the player to adjust their image of the PC varies depending on how much they project themselves into it and how much they are willing to actively create interpretations about the characters, which leads to different receptions of unexpected character actions. Finally, we showed how our findings relate to existing theoretical models, such as the player-avatar relationship from the genre of MMORPG and the concept of alterbiographies.

Our study has several limitations. Due to the relatively small sample and exploratory nature of the study, the depth of insights for each variable of the PC relationship is limited and provides a great avenue for future inquiry. In addition, while we tried to balance the opinions about the game in our participant sample, it might be slightly biased toward the positive, due to the possible reluctance of disappointed players to take part in an interview. Furthermore, as the first author did not code all transcripts herself there might be a loss of information, however, she repeatedly consulted all transcripts and discussed with the involved researchers.

There are several opportunities for future research inquiry. Our themes could be applied to other game cases of character switches. For example, a study of *Metal Gear Solid 2* (Konami, 2001) could show how despite the game receiving high appraisals for the gameplay and story, the character switch was rather controversial. Even though in *Metal Gear Solid 2* the switch is not from protagonist to antagonist, there seems a varying tolerance of the character switch and there are players who strongly reject an intrusion into their agency of controlling their favorite character^4^. Further, the flexibility of the character attachment seems different among players, since in some positive reviews the new character is readily accepted (“He”s ok with me.”^5^) and in some positive and most negative reviews the character is rejected (“[he] truly destroys this game.”^6^), mentioning his non-attractive and contrasting personality in comparison to the original “cool” PC. This could imply variances in how those players are attached to their PC. Finally, differences in the malleability of the character image could be seen in how the negative reviews complained about how “confusing^7^” and “ridiculous^8^” the story is, hinting at difficulties adjusting to changes in the story and character image. Such tentative observations could be investigated through future in-depth analyses.

Our exploratory qualitative work can also be extended with quantitative studies on each dimension lined out by the three themes presented in this study. A quantitative inquiry will verify or disprove the current findings and provide stronger evidence for the proposed themes. For example, concerning the flexibility of character attachment, a survey using measures of character attachment and the perception of sociality of the PC can provide more solid evidence for the relation between different kinds of character attachments and game satisfaction. Cross-genre studies could further help to position player-character relationships in narrative games within the broader context of gaming experiences.

## Data Availability Statement

The datasets presented in this article are not readily available because participants did not agree to share their private interview data to the public. Requests to access the datasets should be directed to Young Yim Doh, yydoh@kaist.ac.kr.

## Ethics Statement

This study involving human participants was reviewed and approved by the KAIST Institutional Review Board. The participants provided their written informed consent to participate in this study.

## Author Contributions

VE conducted, transcribed, and coded all interviews in English, coded one interview in Korean, and wrote the manuscript. SL conducted all interviews in Korean, coded two Korean interviews and revised the manuscript. YYD supervised the work, consulted the research process, and revised the manuscript. All authors participated in the conceptualization and design of this research and the iterative creation of the themes.

## Funding

This research is supported by Year 2020 Culture Technology R&D Program by Ministry of Culture, Sports and Tourism and Korea Creative Content Agency (Project Name: Research Talent Training Program for Emerging Technologies in Games, Project Number: R2020040211).

## Conflict of Interest

The authors declare that the research was conducted in the absence of any commercial or financial relationships that could be construed as a potential conflict of interest.

## Publisher's Note

All claims expressed in this article are solely those of the authors and do not necessarily represent those of their affiliated organizations, or those of the publisher, the editors and the reviewers. Any product that may be evaluated in this article, or claim that may be made by its manufacturer, is not guaranteed or endorsed by the publisher.

## Footnotes

^1^ https://www.zoom.us/

^2^ www.metacritic.com

^3^https://www.metacritic.com/game/playstation-4/the-last-of-us-part-ii/user-reviews?dist=positive (accessed April 13, 2021).

^4^ https://www.metacritic.com/game/playstation-2/metal-gear-solid-2-sons-of-liberty/user-reviews?dist=negative

^5^https://www.metacritic.com/game/playstation-2/metal-gear-solid-2-sons-of-liberty/user-reviews?dist=positive, Ivanm. Nov 15, 2009.

^6^https://www.metacritic.com/game/playstation-2/metal-gear-solid-2-sons-of-liberty/user-reviews?dist=negative, Kyle B. Nov 14, 2002.

^7^https://www.metacritic.com/game/playstation-2/metal-gear-solid-2-sons-of-liberty/user-reviews?dist=negative, DeanR. Dec 29, 2008.

^8^https://www.metacritic.com/game/playstation-2/metal-gear-solid-2-sons-of-liberty/user-reviews?dist=negative, CarlosFromPeru, Aug 4, 2007.
