# Supplementary material for: Player-Character Relationship and Game Satisfaction in Narrative Game: Focus on Player Experience of Character Switch in The Last of Us Part II
Source: Front Psychol. 2021 Sep 27;12:709926. doi: 10.3389/fpsyg.2021.709926 (PMC8503519; doi:10.3389/fpsyg.2021.709926)
Supplement: Supplementary file 1 [file Table_1.DOCX]

Supplementary Material - Interview guide

1. What role has gaming in your life, what games do you like?
2. Why did you choose to play The Last of Us 2?
3. How did you like the game?
4. What do you think about the different player characters, were there some which you liked playing more than others?
5. For each Character Joel, Ellie, Abbie:
   1. Can you describe this character? For instance appearance, personality, role or function of this character.
   2. How did you like this character, how did you like playing as him/her?
   3. How would you describe your relationship to that character? For example, do you think the character is your friend, yourself, your daughter, your enemy?
   4. What did you think about the skills and abilities while playing this character?
   5. Were there any specific moments which you particularly liked or disliked in the game? What were your thoughts and feelings at that moment?
   6. Did something change in the way you think about the characters? (During the game and after)
6. Why do you think you feel this way for that character?
7. Can you tell us about some impressive player characters in other games?
